# *FUNCTION* is now functional

**DOI:** 10.1093/function/zqaa001

**Published:** 2020-05-05

**Authors:** Ole H Petersen

**Affiliations:** School of Biosciences, Cardiff University

It is a great pleasure to announce that the American Physiological Society’s new peer-reviewed open access journal *FUNCTION*, published in partnership with Oxford University Press, is now operational and ready to receive submissions. *FUNCTION* will provide a multidisciplinary home for cutting-edge research describing major advances in basic and translational science that significantly broaden the understanding of biological function.

*FUNCTION* aims high and we have therefore assembled an extraordinary team of eminent executive editors, consulting editors, and editorial board members. A substantial number (19) are Fellows/Members of the Royal Society, the National Academy of Sciences, the German National Academy of Sciences Leopoldina, and/or Academia Europaea and includes three Nobel Laureates. However, we also have many younger rapidly rising stars. At the beginning of its life, the editorial team is clearly *FUNCTION*’s greatest asset. Through the editorial team’s leadership, *FUNCTION* will accelerate discovery and move the field of physiology forward in the years to come.

Why do we need this new journal? One important reason is the movement toward open access and, more recently, the demand from many leading funding organizations worldwide for grantees only to publish in full and immediate open access journals. I must confess that it was only in September 2018 that I was fully converted to this cause. At a dinner in London, following the celebration at the Royal Society of the 30th anniversary of the Academia Europaea, I was seated next to Robert-Jan Smits, one of *Nature*’s “top 10” in 2018, who had just received the Academy’s Gold Medal. During his exceptional tenure as the EU Commission’s powerful Director-General for Research and Innovation, Robert-Jan Smits was responsible for the creation of the European Research Council (ERC), Horizon 2020, and later the Commission’s Scientific Advice Mechanism. However, at the time of this dinner, he was the Commission’s Special Envoy for Plan S, demanding that the results of research funded by public grants must be published in open access journals. We talked extensively that night about the future of scientific journals. I was impressed by Robert-Jan Smits’s enthusiasm and his clear vision of a world in which all real knowledge would be readily and freely accessible to everyone. I also understood that the world of science publishing is undergoing a transformation and the time for launching a high-profile open access journal in the field of physiology is now.

Our vision is for *FUNCTION* to be the home for the best of physiology and pathophysiology or, in other words, function and malfunction. As the inaugural Chair of the ERC’s starting grant panel for physiology, pathophysiology, and endocrinology (2009–2011), I saw that the vast majority of grant proposals were in the area of pathophysiology. This was not for tactical reasons, because the ERC assesses proposals exclusively on the basis of their intrinsic quality and without any regard for potential utility. The reason is that there are massive opportunities in this area, as physiologists have developed a sophisticated knowledge of the basic functions of the body and a fantastic technical armory, that now allow us to effectively explore the pathophysiology of many important diseases. I, therefore, expect that a substantial fraction of the publications in *FUNCTION* will deal with pathophysiology. *FUNCTION* will not be a clinical journal; yet will welcome clinical and translational perspectives on important new pathophysiological findings.

Editing a scientific journal is a mixture of science and art. A good editor cannot simply act as a post-box distributing articles to specialist reviewers and then follow the advice of a majority. The advice of specialist reviewers is essential, but their arguments have to be interpreted and weighed by the editor. It is always easy to find reasons for not publishing a paper, but perhaps it is more important to recognize a real breakthrough in an imperfect article. In my experience, it is essential to remember that the most critical referee is not necessarily right.

Articles that are scientifically important and sound deserve to be presented in such a way that they become accessible to a broad audience. This was brought home to me long ago by a personal experience. In 1983, Peter Newmark, who was at that time *Nature*’s biological sciences editor, and later started *Current Biology*, invited me to write a review article on calcium-activated potassium channels and secretion. I wrote the paper in the summer of 1983 and it became not only a review, but also incorporated a new model for control of fluid secretion. After peer review and correction of proofs, I expected rapid publication, but I received instead a telephone call from *Nature*’s formidable and rather intimidating Editor-in-Chief, John Maddox. He told me that he had just read my article and although he found the paper interesting, he was now going to rewrite it. Knowing that John Maddox was a physicist, this sounded frightening to me, but there was of course nothing I could do about it. Shortly thereafter, I received the heavily edited version of my article, which now contained numerous and rather serious errors. However, the language was much more vivid and there was a much-improved narrative flow, so the article had become much more readable for a nonspecialist audience. I corrected all the errors and the article, which eventually became a Science Citation Classic, was duly published in *Nature* in February 1984. Many years later, in 2000, both John Maddox (now Sir John) and I were elected Fellows of the Royal Society and I was seated next to John Maddox at the celebratory dinner. I recollected my experience from 1983/1984 and John Maddox revealed that he had the habit of reading and correcting the whole front part of the journal prior to publication. He wanted to ensure optimal clarity and a logical flow as well as removal of all specialist jargon. From this, and later work as a member of the editorial board of *Current Biology* (2002–2017), I learned a lot about writing and editing.

A new journal should have innovative features and *FUNCTION* has several of these. A good scientific journal should be much more than a database of new findings. In the biomedical sciences, we need context and perspective and *FUNCTION* will endeavor to provide this in relation to every single original paper we publish. We will commission a perspective article from a leading expert as soon as an original research paper has been accepted. I also believe that we have a duty, to those who entrust their best scientific papers to us, to ensure that their new findings get maximal exposure by providing platforms, such as *FUNCTION* symposia and American Physiological Society meetings, for presentations by authors of papers published in *FUNCTION*. We plan to invite *FUNCTION* authors to share their discoveries at meetings and we plan to solicit speakers to contribute to *FUNCTION*.

During my many years in science, I have noted that there has been an increasing tendency for top journals to demand more and more data. Often long lists of required additional experiments are produced by reviewers—the so-called “reviewer experiments”—and it is implicit in the demand for revision that if these experiments do not work out as predicted, the paper will be rejected. Given that a paper in a top journal can be career changing, the temptation to exclude data that do not “fit” can be enormous, not to speak of even worse actions. Many consider that this issue has contributed to the problem of irreproducible data. How is *FUNCTION* going to deal with this problem? First of all, we will aim to be totally clear and transparent when we come to a decision at the end of the first review round. If the “story” is interesting and convincing, we shall not demand further experiments. If the “story” is interesting, but there is a clear need for a few further experiments, we shall require these to be carried out. However, we think that if a team has entrusted their work to us we have an obligation to be fair to them and in such a case it will therefore be made clear that the paper will be accepted irrespective of the outcome of the additional experiments. Clearly, in some cases, this may necessitate changes in the discussion and the conclusions. If large numbers of experiments are required in order to make a paper convincing, it will be rejected, but if there is an interesting core, resubmission of an entirely new and more complete paper will be encouraged.

As it increasingly takes longer and longer to produce a high-quality paper, because of the demand for completeness, there is a danger that important new results take too long to reach the scientific community, delaying real progress in the particular field. We are therefore introducing a category of focus paper—known as the Function Focus—that reports a very significant and fully documented single finding, but does not require exploration of all the ramifications that typically are expected for full papers.

Having served as European executive editor of *Physiological Reviews* (2003–2011) and, more recently, senior reviews editor for *The Journal of Physiology* (2016–2019), I fully appreciate the immense value and influence review articles can have, but I am also conscious of significant failings that have evolved over time. Review articles have increasing numbers of references, sometimes massive numbers, and critical statements of supposed facts are often summarized in sentences that end with a long string of references. These are frequently a mixture of other review articles and an assortment of original articles that may or may not include the reference to the original discovery. Sometimes, the only references are to other review articles and when these are consulted it may be seen that they, again, do not refer to original articles, but to other review articles. In too many cases, it turns out to be impossible to find the actual evidence for a particular claim, which may not even exist. This has distorted the literature and this issue is confounded by an increasing tendency also for authors of original papers to quote review articles rather than the articles providing the actual evidence. At a translational medicine meeting in Budapest last year, at which many leading editors spoke, there was concern about this problem, which has generally been “swept under the carpet.”

In my current work for the EU Commission’s Scientific Advice Mechanism, I have overseen the generation of several expert evidence review reports by Science Advice for Policy by European Academies. The evidence review reports summarize the published evidence in a particular area of concern and assess the relative reliability of the key findings, but do not express opinions. These reports are then used as the basis for a report called “scientific opinion,” which is produced by a separate group of chief scientific advisors to the EU Commission. It occurred to me that such a clear separation of fact and opinion could also be helpful in scientific publications. *FUNCTION* will therefore introduce a new type of Evidence Review, in which there will be no references to other review articles but only to the original literature. Furthermore, precise referencing—making explicit which original article contains which specific piece of information—will be encouraged, so that the ambiguity in many current review articles with regard to “who did what and when” will be avoided. This same principle of precise referencing can, and will, also be applied to the introduction and discussion sections of original papers to be published in *FUNCTION*.

*FUNCTION* is now reaching out to the physiological and pathophysiological communities and asks you to join us in what we think will be a very exciting venture. *FUNCTION* looks forward to working with you to publish your best work in the most attractive way possible and give it the context and prominence it deserves. We are waiting for your submissions of original papers as well as Focus articles and will be happy to consider proposals for evidence reviews.

